# Robust and accurate pulmonary nodule detection with self-supervised feature learning on domain adaptation

**DOI:** 10.3389/fradi.2022.1041518

**Published:** 2022-12-15

**Authors:** Jingya Liu, Liangliang Cao, Oguz Akin, Yingli Tian

**Affiliations:** ^1^ The City College of New York, New York, NY, USA; ^2^ UMass CI, Amherst, MA, USA; ^3^ Memorial Sloan Kettering Cancer Center, New York, NY, USA

**Keywords:** self-supervised learning, lung nodule detection, false positive reduction, feature pyramid network, medical image analysis, deep learning, feature learning

## Abstract

Medical imaging data annotation is expensive and time-consuming. Supervised deep learning approaches may encounter overfitting if trained with limited medical data, and further affect the robustness of computer-aided diagnosis (CAD) on CT scans collected by various scanner vendors. Additionally, the high false-positive rate in automatic lung nodule detection methods prevents their applications in daily clinical routine diagnosis. To tackle these issues, we first introduce a novel self-learning schema to train a pre-trained model by learning rich feature representatives from large-scale unlabeled data without extra annotation, which guarantees a consistent detection performance over novel datasets. Then, a 3D feature pyramid network (*3DFPN*) is proposed for high-sensitivity nodule detection by extracting multi-scale features, where the weights of the backbone network are initialized by the pre-trained model and then fine-tuned in a supervised manner. Further, a High Sensitivity and Specificity (*HS${}^{2}$*) network is proposed to reduce false positives by tracking the appearance changes among continuous CT slices on Location History Images (LHI) for the detected nodule candidates. The proposed method’s performance and robustness are evaluated on several publicly available datasets, including LUNA16, SPIE-AAPM, LungTIME, and HMS. Our proposed detector achieves the state-of-the-art result of 90.6% sensitivity at 1/8 false positive per scan on the LUNA16 dataset. The proposed framework’s generalizability has been evaluated on three additional datasets (i.e., SPIE-AAPM, LungTIME, and HMS) captured by different types of CT scanners.

## 1. Introduction

Lung cancer is one of the world’s leading cancers in terms of incidence and mortality rates (1). At the time of diagnosis, the disease stage is closely related to the survival of patients with lung cancer. Therefore, it is critical for the efforts to identify and intervene in lung cancer in the early stage (2). Computed tomography (CT) has been shown to visualize tumors better in early clinical diagnosis (3). However, it is cumbersome and time-consuming for radiologists to detect and label tumors on CTs manually. To better assist the diagnosis of lung cancer, CT-based automatic pulmonary nodule detection methods have been widely explored (4–9) to develop computer-aided diagnosis (CAD) systems (10,11). The CAD framework for nodule detection commonly consists of a nodule detector identifying and detecting the location of nodule candidates and a classifier further distinguishing the false detected candidates from true nodules with a false positive reduction procedure. In recent years, deep learning based methods demonstrated excellent performance on medical image analysis and preliminary work of lung nodule detection (12–16) yielded a high sensitivity of over 95% on LUNA16 challenge dataset (17), but at a high false positive rate (i.e., 8 false positives per scan) which limited their uses in real clinical processes. Reducing the false-positive rate remains an open question. Most of the existing methods achieved sensitivities below 75% at 1/8 false positive per scan. The high false-positive rate is mainly caused by the following two reasons. (1) Some normal tissues are morphologically similar to the nodules in the CT image, leading to a high false detection rate. The approach for differentiating between the tissue and nodule is very crucial to reduce false positives for automatic lung nodule detection scheme. (2) The volumes of nodules significantly differ from the total CT volume, which may lead to the miss detection of some nodules. For example, in the LUNA16 dataset (17), nodules size can range from 3 mm to 30 mm (diameter), which varies up to 10 times. Only 0.059% of the total CT scan volume is occupied by a 10 mm nodule in diameter on a CT scan in a resolution of 213×293 pixels and 281 slices. Therefore, it is essential to design methods for detecting small nodules from large CT scan volume and further distinguish the normal tissues with similar appearances of nodules for CT scans with various machine settings and intensity scales.

For a data-dependent deep learning-based framework, artifacts such as intensity, machine setting, machine noise, and image protocol for collected CT scans could cause systematical differences. In order to develop robust deep learning-based lung nodule detection methods to handle CTs collected by different vendors of CT scanners, there is a need for a large amount of training data to be labeled. However, manually annotating a large number of CT scans can be tedious, attention-demanding, and time-consuming. It also requires human expertise in the specialty of radiology. Recently, a self-supervised learning approach (18,19) is proposed to learn the intermediate representation from the sizeable unlabeled dataset by a well-designed pretext task in a supervised learning manner. Inspired by the rotation ConvNets (20,21), in this paper, we simply rotate the CT scan at certain angles and design a rotation classification network as the pretext task to distinguish the rotation angle of each CT scan. The well-trained model effectively learns the rich features and semantic concept of the CT scans from a large unlabeled dataset and is then further applied as a pre-trained model of the nodule detector for robust nodule detection training on small annotated datasets.

**Figure 1:**
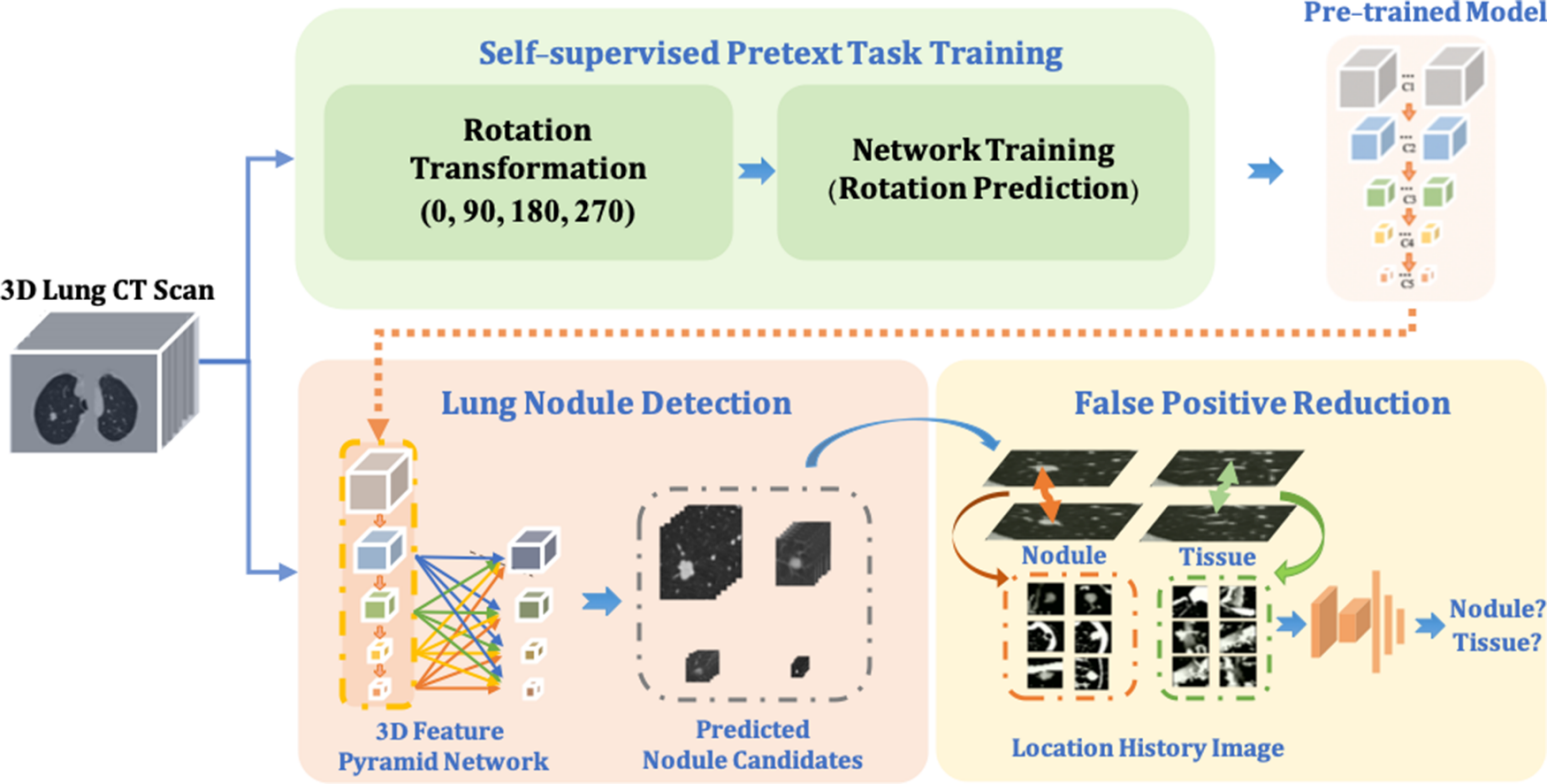
The proposed robust nodule detection framework *3DFPN-HS${}^{2}$* consists of 3D Feature Pyramid ConvNet (*3DFPN*) as lung nodule detector and *HS${}^{2}$* as false positive reduction network for high sensitivity and specificity lung nodule detection. (1) To improve the robustness of the framework across different datasets, a pre-trained model trained on the backbone network of detector *ResNet-18* is applied to the nodule detection network. The pre-trained model is obtained by a simple yet effective pretext task training through a rotation prediction network. The original CTs are rotated through a geometric transformation at (0, 90, 180, 270) degrees and followed by a classification network to predict the rotated angles of CT scans. (2) The *3DFPN* takes the input of the entire CT scan to predict nodule candidates. The backbone network (*ResNet-18*) of *3DFPN* is initialized by the weights from the pretrained model and then is fine-tuned with small datasets with annotation for pulmonary nodule detection in a supervised schema. (3) For the detected nodule candidates, the *HS${}^{2}$* network eliminates the false prediction of normal tissues based on the change in position of the continuous CT slices on LHI images. The detailed structure of self-supervised pretext task training is shown in Figure 2, the proposed *3DFPN* network can be found in Figure 3, and LHI is illustrated in Figure 4.

In this paper, as shown in Figure 1, our proposed framework contains three main components. (1) To improve the robustness of the nodule detector across datasets without requiring extra annotations, a self- pre-trained model is proposed to learn the rich spatial features among CT scans obtained from various manufacturers and obtained through the training by applying rotation prediction as pretext task. (2) Motivated by 2D Feature Pyramid Network (*FPN*) (22), a 3D high-sensitivity feature pyramid network (*3DFPN*) is developed for multi-scale feature prediction by combining the low-level high-resolution feature with high-level semantic features for the nodules with different sizes. (3) A false positive reduction network based on location history images to distinguish differences in the spatial distribution of nodules and normal tissues in continuous CT slices significantly eliminates the false positives while maintaining high sensitivity and specificity.

We proposed an accurate and robust pulmonary nodule detection framework (*3DFPN-HS${}^{2}$*) by integrating an accurate nodule detection model with a novel false positive reduction method to achieve the high sensitivity and specificity of diagnosis. This paper is an extension of our preliminary work (23), and the new contributions are summarized as follows:

1. To improve the robustness and generality of the nodule detector without additional annotations, we adopted a pre-trained model that can significantly improve the performance of the model across datasets with a simple and effective self-supervised learning schema.
2. By combining the pre-trained model with the two-stage framework (*3DFPN-HS${}^{2}$*), the experiments and results on the LUNA16 dataset demonstrate state-of-the-art performance, especially at low false-positive rates.
3. The generalizability of the proposed framework has been evaluated on three additional small datasets (SPIE-AAPM, LungTIME, and HMS) captured by different types of CT scanners show the robustness of the proposed framework, which has a high potential for application in clinical practice.

The remainder of this paper is organized as follows. Section 2 introduces the related work on self-supervised feature learning, object detection, and lung nodule detection from CT scans. Section 3 explains the proposed method. Section 4 presents the implementation details, experimental results, and discussions. Finally, Section 5 summarizes the remarks of this paper.

## 2. Related work

As data-driven computational mechanisms, supervised convolutional neural networks (ConvNets) usually require large-scale labeled data to obtain good performance and overcome overfitting. Manually labeling a large number of CT scans is very expensive and requires multiple expert radiologists to perform the task to address reader agreement and variability issues. Therefore, in computer vision, some researchers proposed self- or un-supervised learning methods to learn feature representations without requiring manual data annotations (24–28). The intermediate representations of images and videos are learned by training the networks on one or multiple pretext tasks (e.g., regression or classification) with the modification of unlabeled data.

Recently, self-supervised learning methods are widely explored, and various pretext tasks have been proposed with learning by distinguishing the distorted transformations (18), adopting patches to predict relative position (29), colorizing to map the image to a distribution (30), and distinguishing jigsaw puzzle with shuffle patches (19). Zhuang *et al.* (31) proposed a Rubik’s cube task which extended jigsaw puzzle (19) of re-ordering 2D image patches to rotate and re-order 3D cubes. The result demonstrated the performance improvement for classification and segmentation tasks on CTs. Zhou *et al.* (32) proposed model genesis as the pretext task training through image distortion, in-painting, and unified method and have proven to benefit the downstream tasks on image classification and segmentation without any annotation. Jing and Tian reviewed self-supervised learning methods in the comprehensive survey paper (33). The previous work of self-supervised learning related to lung nodule mainly focuses on nodule classification and segmentation. In this paper, we aim to demonstrate the robustness of the self-supervised learning method on the pulmonary nodule detection task with the rich semantic features learned from large scale lung CT scans. (20) rotated each input image by a multiple of 90 degrees and learned the semantic content of the image through an image rotation prediction network. However, previous methods are based on two dimensions and lacked spatial information. Recently, Jing and Tian (21) designed a rotation transformation network for a 3D input sequence to learn rich features from the video. The network can learn high-level spatial information of objects in videos while predicting the correct rotations. Following the framework in (21), by treating each CT scan as a video, we employ simple yet effective rotation prediction pretext task for predicting the rotation angle of 3D CT scans to obtain rich spatial information of CT scans. Several deep learning-based frameworks for object detection are proposed to handle small-scale and multi-scale objects (34,35). Single Shot multi-box Detector (*SSD*) (36) applied the pyramid feature hierarchy in the deep convolutional network, which directly detected multi-scale objects by using multi-layer feature mapping in a single pass. However, *SSD* cannot reuse low-level feature maps that cause the miss detection of small objects. In order to detect small objects, Scale Normalization for Image Pyramids (*SNIP*) (35) selectively back-propagated the gradient of objects in different scales. Although small object detection performance has been significantly improved,the computation cost could be very high by applying multiple images as input. To date, a 2D Feature Pyramid Network (*FPN*) (22) demonstrated the effectiveness of small object detection by extracting the multi-scale feature maps containing the general low-level features of objects at different scales. A top-down path was introduced to pass global context information through lateral connections of high-level and low-level features. The computation of feature extraction was reduced by directly applying multi-scale feature maps. This *FPN* framework can be applied to lung nodule detection on each 2D CT slice. However, without 3D information among CT slices, high false positives were produced.

Continuous efforts have been made to detect pulmonary nodules with CT scans. Compared with the traditional methods based on intensity, shape, texture features, and context features, the deep learning-based methods have shown significant performance improvements (37–40). (16) could achieve an average sensitivity of 84.2% by a *3D Faster R-CNN* detector to learn the rich nodule features combined with a dual-path network and an encoder-decoder structure without false positive reduction, while Single-Shot Single-Scale Lung Nodule Detection (*S4ND*) (14) introduced 3D dense connections and investigated a down-sampling method for small nodule detection. These frameworks employed only a single scale feature map and were limited in the detection of nodules with a large range of sizes. (41) proposed a multi-scale nodule detection method. The method firstly segmented the lung boundary delineation to obtain the lung region and then applied three sub-algorithms to detect the candidates in the three nodule size intervals. Although nodules of different sizes were treated separately, the sensitivity to 85.6% at 8 false positives per scan was limited by the rule-based threshold and morphological algorithms.

Furthermore, in order to reduce false positives, Dou et al. (13,42) applied three different 3D ConvNet architectures to adapt the different scales of nodules, and manually set the threshold to combine the weights. Ding et al. (12) applied a framework of *2D Faster R-CNN* nodule detector with a false-positive reduction classifier and obtained 89.1% average sensitivity. Multi-scale Gradual Integration Convolutional Neural Network (*MGI-CNN*) (43) used the image pyramid network with a multi-stream feature integration for small nodule detection and false-positive reduction. However, the computation cost of these frameworks is expensive due to the great effort to extract feature maps from images in different sizes and the multiple training process. Wang et al. (15) applied a *2DFPN* network for lung nodule detection, followed by a Conditional 3-Dimensional Non-Maximum Suppression (Conditional *3D-NMS*) and Attention 3D CNN (Attention 3D-CNN) for false-positive reduction. However, without the spatial features within continuous CT slices, the high false-positive candidates were introduced, resulting in great efforts in the reduction process.

In this paper, we propose a rich spatial feature extraction method, an accurate multi-scale nodule detection network, and an efficient false-positive reduction algorithm for accurate and robust pulmonary nodule detection.

## 3. Method

In this section, we describe the details of the proposed accurate and robust pulmonary nodule detection framework. As shown in Figure 1, a pre-trained model is firstly obtained by employing the rotation prediction as the pretext task to extract rich spatial features and leverage the noises of CT scans captured by different manufacturers. The weights of the pre-trained model are applied to initialize the backbone network (*ResNet-18*) of the lung nodule detector *3DFPN*. The *3DFPN* takes an entire 3D volume of CT scan as input and outputs the 3D locations of lung nodule candidates. Then, the high sensitivity and specificity (*HS${}^{2}$*) network predicts the probability of the true or false positive for the cropped 3D cube centered with the candidate nodules.

### 3.1. Self-supervised pre-trained model

**Figure 2:**
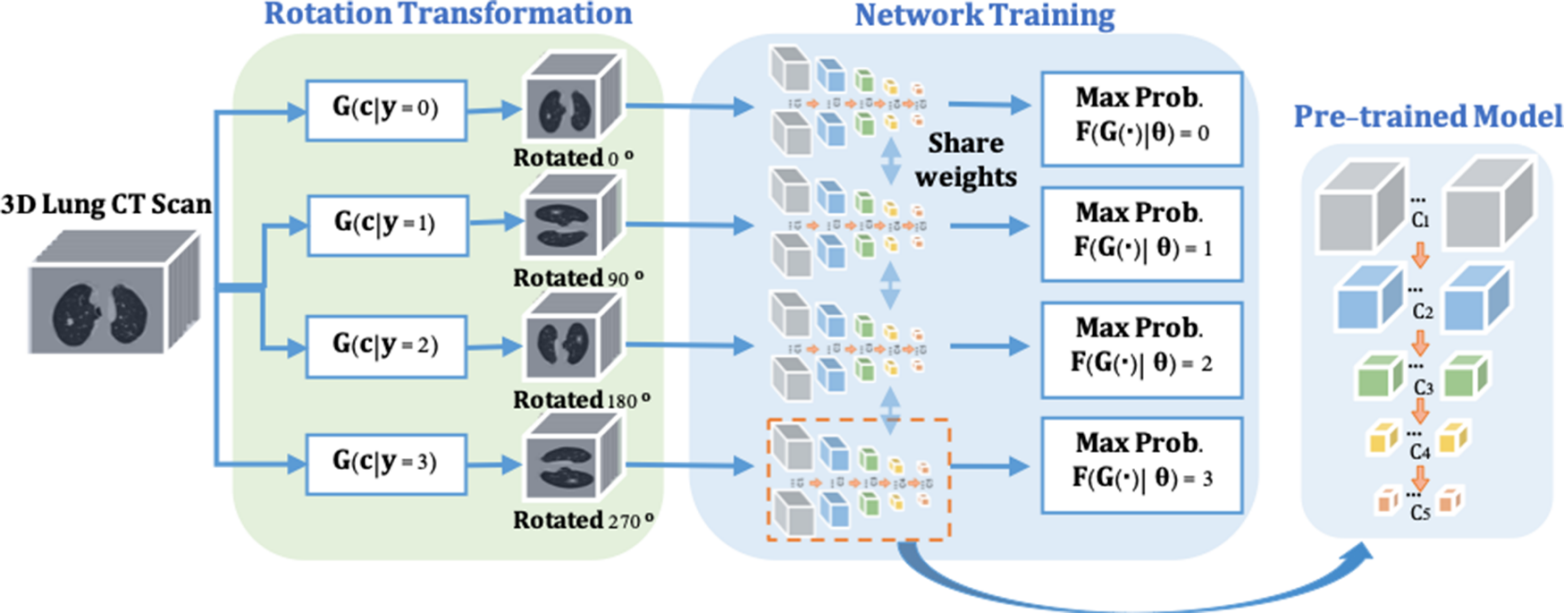
The pre-trained model training consists of two steps. (1) Rotate on input 3D CT scans with four angles as $0^{\circ },90^{\circ },180^{\circ },270^{\circ }$ by the rotation transformation network. (2) The rotation prediction pretext tasks uses the backbone network (*ResNet-18*) of the proposed nodule detector (*3DFPN*) for feature extraction and 2 fully connected (FC) layers to obtain the maximum rotation prediction probability.

To enable the proposed nodule detector for rich 3D CT feature exaction, a pre-trained model is obtained for the representative and discriminative features without using any additional labels. As shown in Figure 2, inspired by (20,21), a rotation transformation is first performed on 3D CT scans to obtain the rotation class with a certain angle. The rotation transform rotates 3D CT scans on the axial plane at an angle θ ($0^{\circ },90^{\circ },180^{\circ },270^{\circ }$). Predicting the correct rotation transformation of an image requires localizing orientations and types of salient objects. Classifying the rotation translation enables ConvNet to learn high-level spatial information of the objects. A dataset consisting of four rotating classes on CT scans is prepared for the pretext task (rotation prediction) training in a supervised manner, aiming to maximize the classification probability of rotation angle. The backbone network of nodule detector (*ResNet-18*) is applied to classify the rotation classes of the input CT scans, followed by two fully connected layers for probability prediction. Therefore, rich spatial CT scans features are learned by distinguishing the feature structures of the lung area in the CT scans. The cross-entropy loss is applied with K rotation angles (here k=4) and rotation r as shown in Equation (1):

(1) $$loss(c_{i}\;|\;\theta )=-1/K\sum_{r=1}^{K}\log(F(G(c_{i},r)\;|\;\theta )),$$

where the classification network is defined as F(⋅|θ) for spatial feature learning and the rotation transformation from the input 3D CT scans to the categories of rotation angle is represented as G(c|y).

### 3.2. 3D feature pyramid network for nodule detection

The recent progress of computer vision suggests feature pyramid networks (*FPN*) for the powerful detection performance on objects at various scales (22). However, the original *FPNs* are designed to handle 2D images. Motivated by this, we propose a *3DFPN* for 3D pulmonary nodule location detection from 3D CT volumetric scans. Different than (22), which only concatenating the upper-level features, to further preserve location details and obtain strong semantic features, a dense pyramid network is proposed by integrating the low-level and high-level layers for high-resolution and high-semantic features, respectively. Table 1 highlights the main differences between *2DFPN* and our *3DFPN*.

**Table 1:** Comparison between *2DFPN* (22) and our proposed *3DFPN*. With the 3D input of the proposed network, the feature pyramid layers are parallel connected with all the high and low-level features.

Method	Input 3D volume	Lateral connections	Integrate upper layer	Integrate lower layer	Upsample higher layer	Downsample lower layer
2DFPN (22)	No	√	√	No	√	No
3DFPN (Ours)	√	√	√	√	√	√

**Figure 3:**
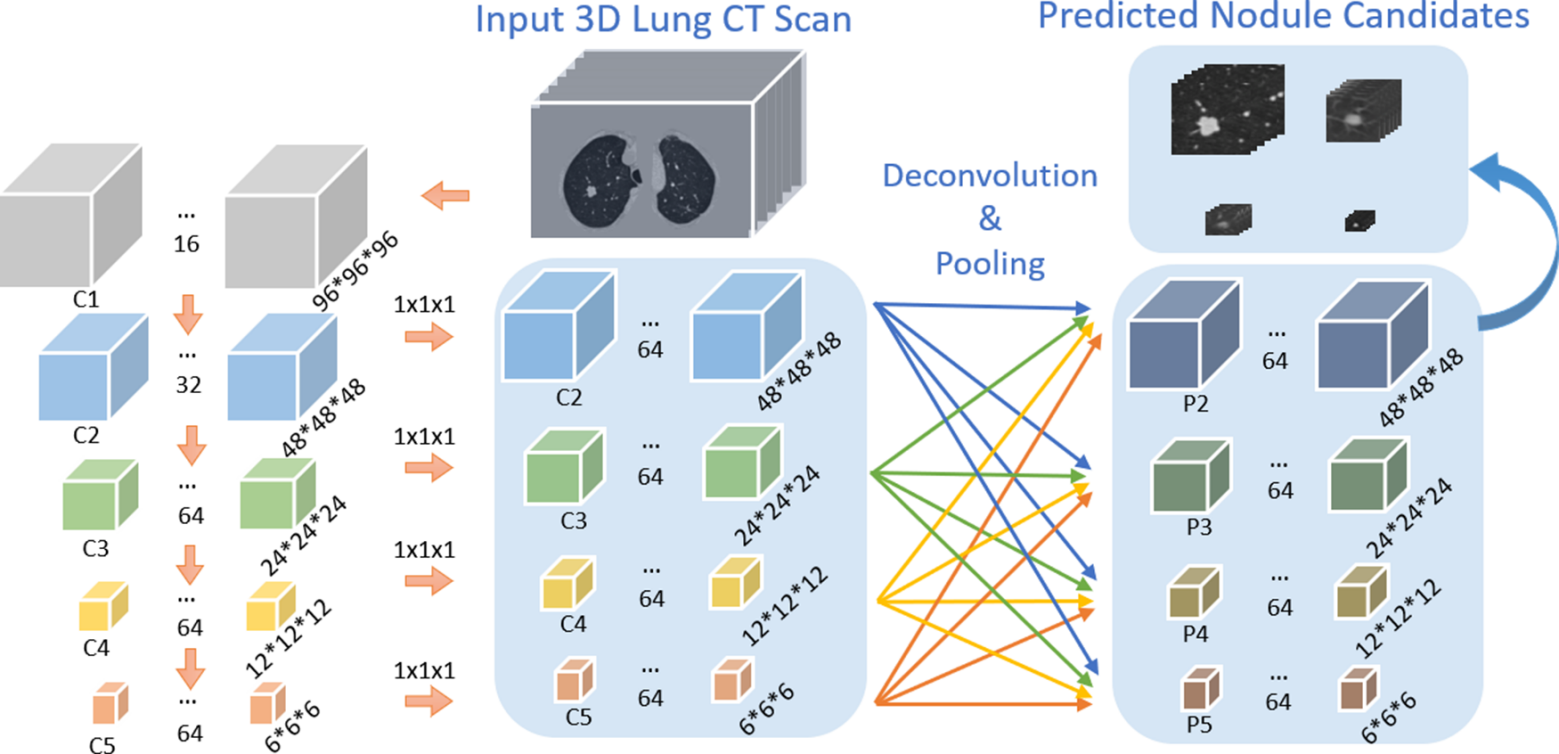
Architecture of our proposed *3DFPN* network. The input 3D volume is split into 96×96 pixels ×96 slices. The size of C1, C2, C3, C4, C5 is $96^{3},48^{3},24^{3},12^{3}$, and $6^{3}$ respectively. The following convolution layer with kernel size 1×1×1 converts feature channels to 64 dimensions. 3D deconvolution and max-pooling layers are applied for integrating each of the convolution layers C2, C3, C4, C5 to the pyramid layers P2, P3, P4, P5.

As shown in Figure 3, the bottom-up network extracts features from the convolution layers 2–5, refer to as C2, C3, C4, C5, followed by a convolution layer with kernel size 1×1×1 to convert the features from the convolution layers to the same size. The feature pyramid network is composed of four layers: P2, P3, P4, P5. The max-pooling layer integrates low-level layer features with high-level features. *3DFPN* predicts a confidence score and the corresponding location as [x,y,z,d] for each nodule candidate, where [x,y] are the spatial coordinates on each CT slice, z is the index number of the CT slice, d is the diameter of the nodule candidate. The backbone network of the detector is initialized with the weights of the pre-trained model and further refined with small labeled datasets with supervised learning.

### 3.3. HS${}^{2}$ network for false positive reduction

**Figure 4:**
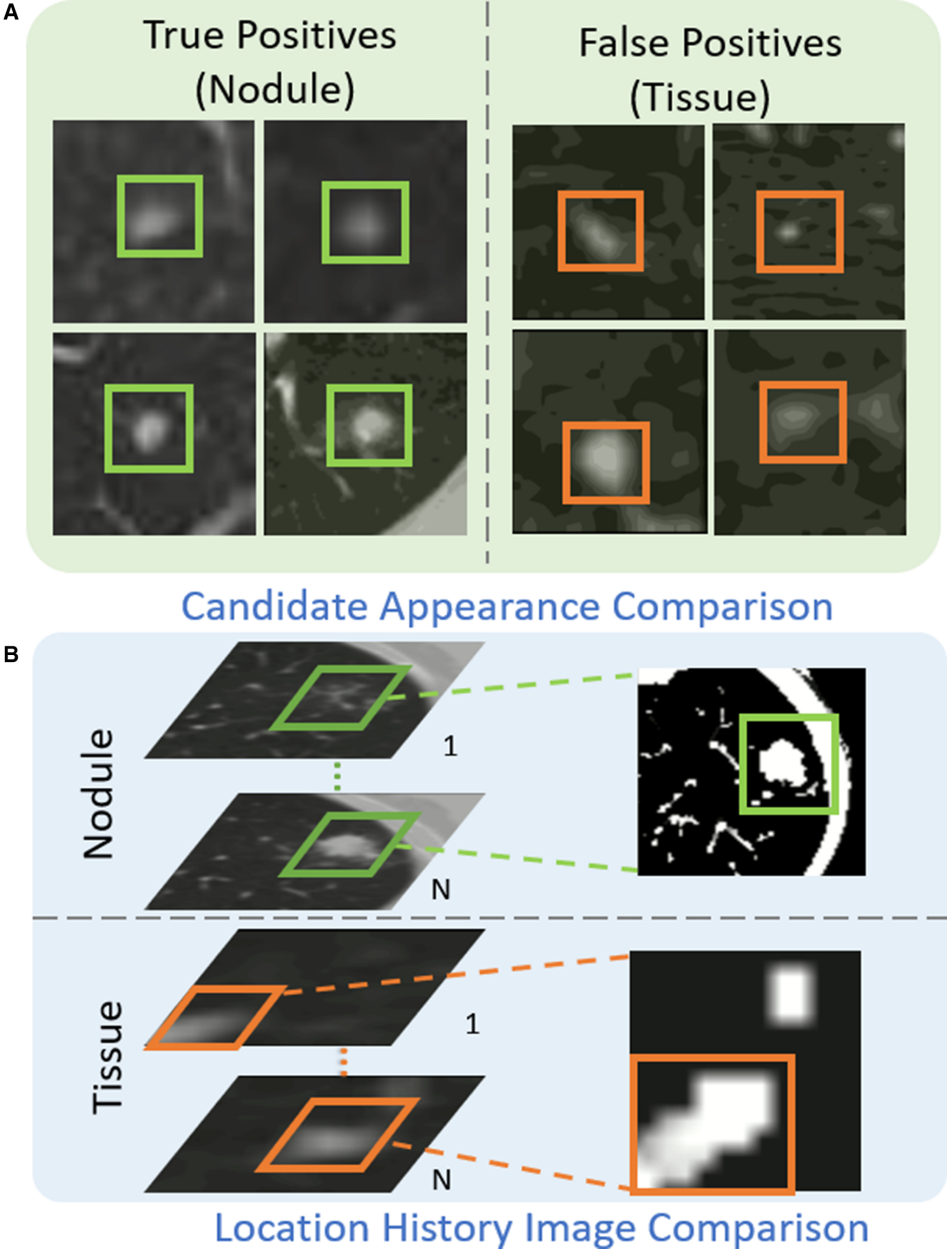
The proposed Location History Images (LHI) to distinguish tissues and nodules from the predicted nodule candidates. (**A**) The true nodules (green boxes) have similar appearances to the false detected tissues (orange boxes). (**B**) The location variances for nodules and tissues are oriented differently in LHIs. True nodules generally have a circular region representing the spatial changes as the brighter center (the size of the nodules decreases in the following CT slides) or a darker center (the size of the nodules increases in the following CT slides). On the other hand, the location variance for false detected tissues tends to change in certain directions, such as a gradual change of the trajectory line.

**Figure 5:**
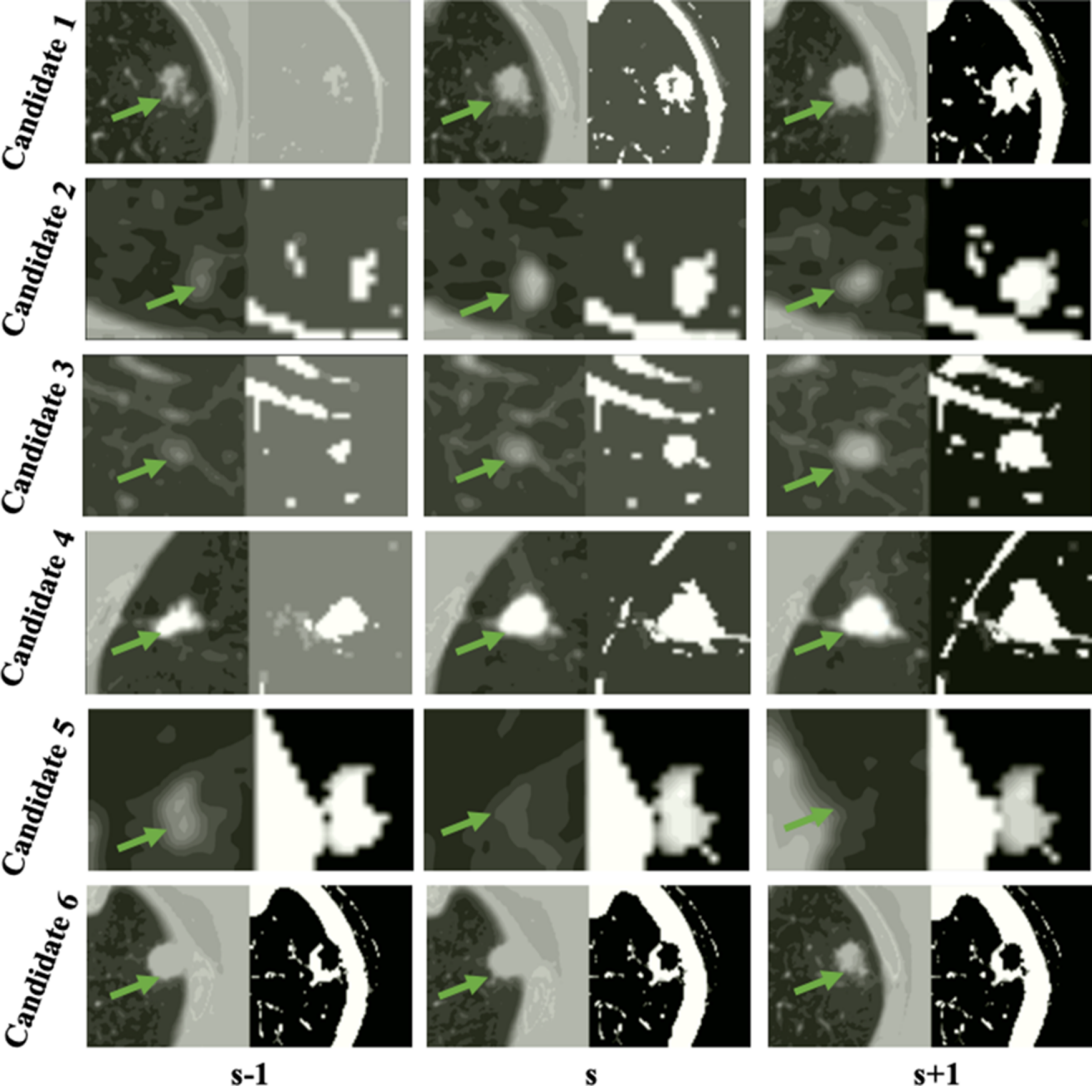
Examples of the detected true nodule candidates (the left image of each column) and their corresponding LHIs (the right image of each column) calculated between (s−2, s−1), (s−1, s), and (s, s+1) slices shown in the s−1, s, s+1 columns. The green arrows mark the position of candidates. As shown in the figure, the true nodules have a circular region on LHI images as the location of the nodule approach to the center or the edge of nodule volume. Furthermore, the center location of the nodule candidates barely changes in the continuous slices.

**Figure 6:**
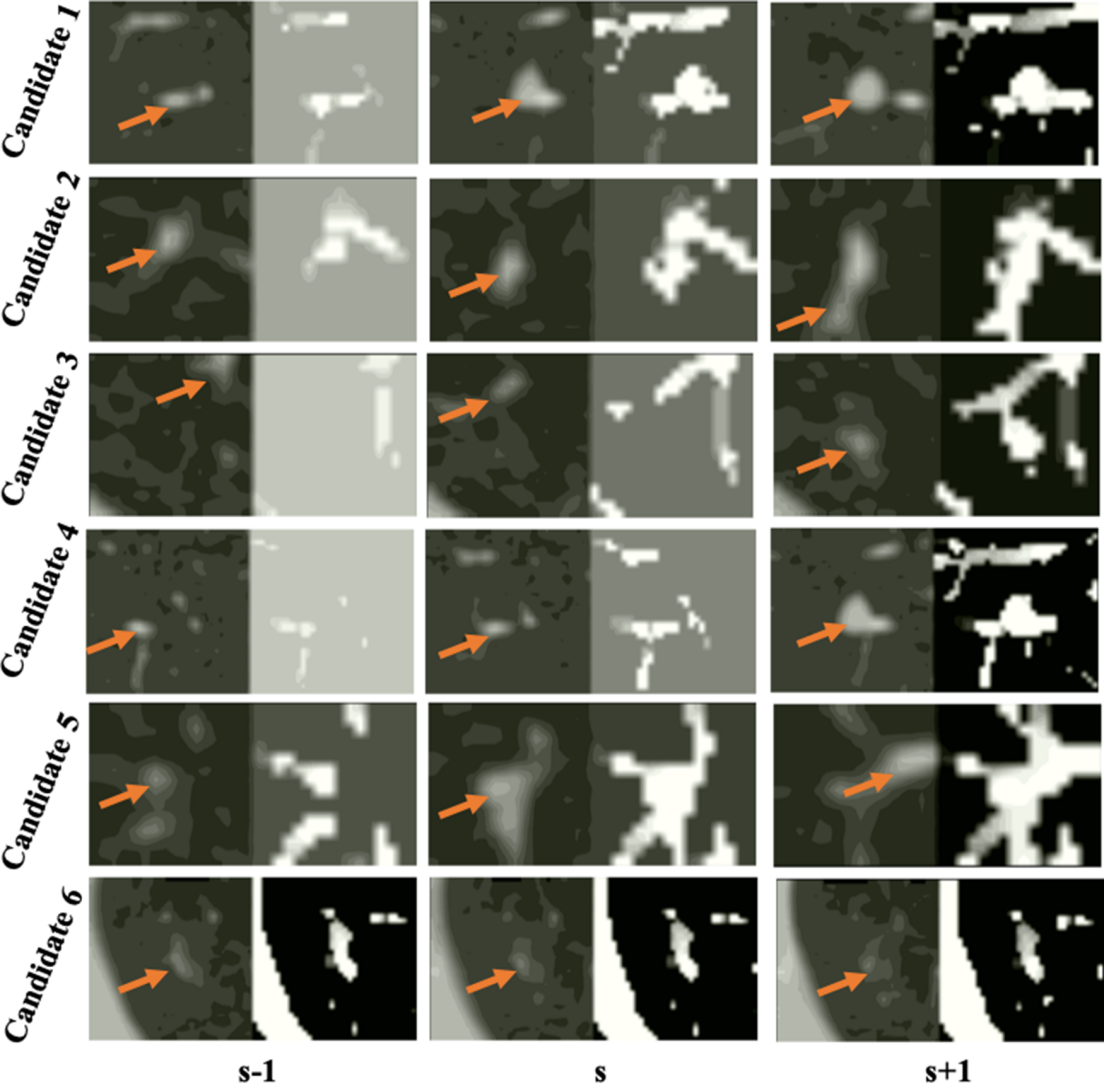
Examples of false detected tissue candidates (the left image of each column) and their corresponding LHIs (the right image of each column) calculated between three continuous slices (s−2, s−1), (s−1, s), and (s, s+1) shown in the s−1, s, s+1 columns. The orange arrows mark the position of false detected tissue candidates. LHIs of tissues are shown to have clear differences with true nodules. Compared with the LHIs of the true nodules in Figure 5, the wide variation of tissue location follows certain patterns, illustrated as intensity variances along the trajectory lines in the LHIs.

As shown in Figure 4(A), the appearance of some tissues (orange box) is similar to that of real nodules (green box), which are likely to be detected as nodule candidates and generate a large number of false positives. Table 2 illustrates the analysis of 300 false positives predicted by the proposed nodule detector *3DFPN*. We observe that 241 False Positives (FPs) are caused by the high appearance similarity of tissues (80.3%), 33 of them are due to inaccurate size detection (11%), and 26 FPs are caused by inaccurate location detection (8.7%). The majority of false positives are caused by normal tissue regions with a similar appearance. However, by treating each CT scan as a video, we discover that the orientations of tissues and nodules present different patterns among the consecutive slices, as shown in Figs. 4(B), 5 and 6. The variance of true nodules tends to expand outward or diminish towards the center in continuous CT slices. Therefore, we propose a novel method to further distinguish the tissues among nodule candidates for false positive reduction.

**Table 2:** Statistic Analysis for False Positive Nodule Candidates.

	Tissue	Inaccurate Size	Inaccurate Location
Percentage	80.3%	11%	8.7%

Inspired by Motion History Image (MHI) (44,45), we define the Location History Image (LHI) as f. The intensity value of LHI within (1,τ) slice is represented by f(x,y,s) by given any pixel location (x,y) on a CT slice s. The LHI is fed to a feed-forward neural network *HS${}^{2}$* with two convolutional layers and three fully connected layers. The *HS${}^{2}$* network refines predicted labels for true nodules and tissues.

The intensity of LHI is calculated according to Equation (2):

(2) $$f(x,y,s)=\left\{ \begin{array}{cc} \tau & \mathrm{if}\;\psi (x,y,s)=1\\ \max(0,f(x,y,s-1)-1) & \mathrm{otherwise} \end{array}\right.,$$

where the update function ψ(x,y,s) is obtained by the spatial differentiation of the pixel intensity of two consecutive CT slices. The algorithm has the following steps. (1) If |I(x,y,s)−I(x,y,s−1)| is larger than a threshold, ψ(x,y,s)=1, otherwise, ψ(x,y,s)=0. (2) For the current slice, if ψ(x,y,s)=1, f=τ. Otherwise, if f(x,y,s) is not zero, it is attenuated with a gradient of 1. If f(x,y,s) equals zero, then remains as zero. (3) Repeat steps (1) and (2) until all the slices are processed. Therefore, the proposed LHIs can adequately represent the location variance among continuous CT slices and their change patterns.

## 4. Experimental results and discussions

### 4.1. Datasets

In this paper, we employ training, testing, and performance evaluation on five public datasets: NLST, LUNA16, SPIE-AAPM, LungTime, and HMS Lung Cancer datasets. Table 3 summarizes the details of these datasets.

**Table 3:** Detailed Information of the Datasets.

Dataset	Manufacturer	CT number	Nodule number
NLST	GE Healthcare Philips Healthcare Siemens Healthcare Toshiba Healthcare	13,762	–
LUNA16	GE Healthcare	888	1186
SPIE-AAPM	Philips Healthcare	70	70
LungTIME	Siemens Healthcare	157	394
HMS Lung Cancer	GE Healthcare	229	254

***NLST** Dataset:* The national lung screening trial (NLST) (46) is a public dataset aimed to determine whether low-dose spiral CT screening for lung cancer can reduce lung cancer mortality in high-risk populations compared with chest screening. The data includes participant characteristics, screening test results, diagnostic procedures, lung cancer, and mortality with more than 75,000 CT scans captured by four different manufacturers of CT scanners (i.e., GE, Philips, Siemens, and Toshiba.) Since this dataset is vast and there are no annotations for nodule locations, the NLST dataset is used for rotation transformation based self-supervised feature learning. In a total of 13,762 CT scans are applied for pretext task training.

***LUNA16** Dataset:* LUNA16 challenge dataset (17) contains 1,186 nodules, ranging in size from 3 to 30 mm from 888 CT scans and agreed by at least 3 out of 4 radiologists. The dataset is officially divided into 10 subsets. To conduct a fair comparison with other lung nodule detection methods, we follow the same cross-validation protocol by applying 9 subsets as training and the remaining subset as testing and reporting the average performance. We split 10% of the training data used for validation to monitor the convergence of the training process. The nodule detector *3DFPN* is initialized by the pre-trained model trained on the NLST dataset and fine-tuned on the LUNA16 training subsets and perform the evaluation on the testing subset.

***SPIE-AAPM** Dataset:* The *SPIE-AAPM* dataset is collected for a ’Grand Challenge’ of the diagnostic classification of malignant and benign lung nodule by the international society for optics and photonics (SPIE) with the support of American Association of Physicists in Medicine (AAPM) and the National Cancer Institute (NCI) (47). It contains 70 CT scans from 70 patients, with the annotation of nodule location and the nodule diagnosis categories of benign or malignant. It is applied in our paper for cross-dataset testing.

***Lung TIME** Dataset:* The Lung Test Images from Motol Environment (Lung TIME) is publicly available and contains 157 CT scans with 394 nodules (48). The nodules are in the range of 2–10 mm in diameter. The CT scans annotations of the nodule location are provided. It is employed in our paper for cross-dataset testing.

***HMS Lung Cancer** Dataset:* HMS Lung Cancer dataset (49) contains the CT scans and lung tumor sections generated by clinical care professionals used in competition with 461 patients. HMS contains a total number of 229 CT scans and 254 nodules with the nodule location annotation. It is employed in our paper for cross-dataset testing.

### 4.2. Data preprocessing

A preprocessing procedure is required to original CT scans for accurate nodule detection. First, the masks of the lung regions are extracted by lung region segmentation. The 2D single slice is processed first with a Gaussian filter to remove the fat, water, and kidney background and followed by a 3D connection volume extraction to remove unrelated areas (50). However, it takes 9 to 22 seconds to obtain the mask for each CT scan. To accelerate the processing speed for large datasets, we employ the LGAN method (51) and train the network on 10,000 CT slices for lung mask extraction to speed up the process in an average of 5 seconds per scan. Additionally, CT scans with a practical value of Hounsfield Unit between [−1200,600] are transformed into the gray value of [0,255] by a linear mapping. The spacing (mm/pixel) of CT scans between different patients and machines is various, and the re-sampling is applied to unify the spacing to 1 mm.

### 4.3. Experimental settings

#### 4.3.1. Self-supervised pre-trained model

The 3D CT scans are rotated at four angles ($0^{\circ }$, $90^{\circ }$, $180^{\circ }$, $270^{\circ }$). The backbone network of *3DFPN* (*ResNet-18*) is used to extract rich spatial features from the input CT scans, and two fully connected layers are applied to maximize the probability of rotation classes. The pre-trained model is then employed to initialize the weights for lung nodule detector *3DFPN*. During the training, the learning rate is set to 0.1, decreasing by 1/2 after 70 and 85 epochs, with the weight decay of $5e^{-4}$. The total training includes 100 epochs, and the batch size is set to 16.

#### 4.3.2. 3DFPN

The *3DFPN* network takes the entire CT scan as input and selects the volume of 96×96×96 pixels through the sliding window schema. This size is selected experimentally to ensure that it accommodates the entire nodule even with the largest nodule (approx. 30 mm). In our *3DFPN*, the anchor sizes used to obtain candidate regions from the feature maps are [3${}^{3}$, 5${}^{3}$, 10${}^{3}$, 15${}^{3}$, 20${}^{3}$, 25${}^{3}$, 30${}^{3}$] pixels. The nodule positions are predicted by 3D feature maps of the corresponding anchor regions. During the training process, the regions to the ground-truth regions with an Intersection-over-Union (IoU) threshold less than 0.02 are referred to as negative samples and with the threshold value greater than 0.4 are positive samples. To avoid the similarity between positive and negative samples, the regions between IoU values are ignored. We follow the 2DFPN (22) to predict the nodule candidates with a 3×3 convolutional layer and followed by two 1×1 sibling convolution as classification and regression layer. The classification layer predicts the confidence score of the candidate classes, and the regression layer learns the offset between the region proposals and the ground-truth. Smooth L1 loss (52) and binary cross-entropy loss (BCE-loss) are used for location regression and classification, respectively. The proposals with a probability greater than 0.1 are selected as nodule candidates. Non-maximum suppression is further applied to eliminate multiple predicted nodule candidates.

#### 4.3.3. HS${}^{2}$ network

The *HS${}^{2}$* network consists of two convolution layers with 30 and 50 output channel dimensions, and three fully connected layers with (2048,1024,512) channel dimensions. The ReLU activation is applied after each convolution layer and followed by a batch normalization layer. 11 continuous CT slices are selected for LHI image generation with 5 slices before and after the current slice of nodule candidate. The sizes of the convolution kernel are set based on empirical experiments. Image patches are aligned with each predicted nodule candidate region but twice the size in both the x and y directions. To calculate the intensity of LHIs, the thresholds of spatial difference between two consecutive slices are set to 30 and 40 for data augmentation. LHIs are resized to 48×48 pixels as the input of the *HS${}^{2}$* network. To overcome the unbalanced data candidates, we randomly sample the false-positive candidates with a similar amount of true candidates and apply the data augmentation, including flipping, rotating, and cropping with 0.9 of the original size. In training, the learning rate starts at 0.01 and decreases to 1/10 for every 500 epochs. 2,000 epochs are executed in training. The average prediction time for an entire CT scan is about 0.53 min/scan on one GeForce GTX 1080 GPU using Python 2.7.

### 4.4. Evaluation metrics

The performance is measured by Free-Response Receiver Operating Characteristic (FROC) analysis and Competition Performance Metric (CPM), the same as other methods. Following the LUNA16 challenge evaluation method, the FROC curve plots detection sensitivity and the points on the corresponding false positives curve are obtained by the true positive rate (true positives over the sum of true positives and false negatives) while the false positive rate at 1/4, 1/2, 1, 2, 4, 8 per scan respectively. The CPM score is calculated by averaging the sensitivity of all false-positive levels per scan. Sensitivity is defined as the ratio of true positives divided by the total number of true positives and false negatives. Specificity is the ratio of true negatives over the total number of true negatives and false positives.

### 4.5. Experimental results and analysis

#### 4.5.1. Comparison with other methods

**Figure 7:**
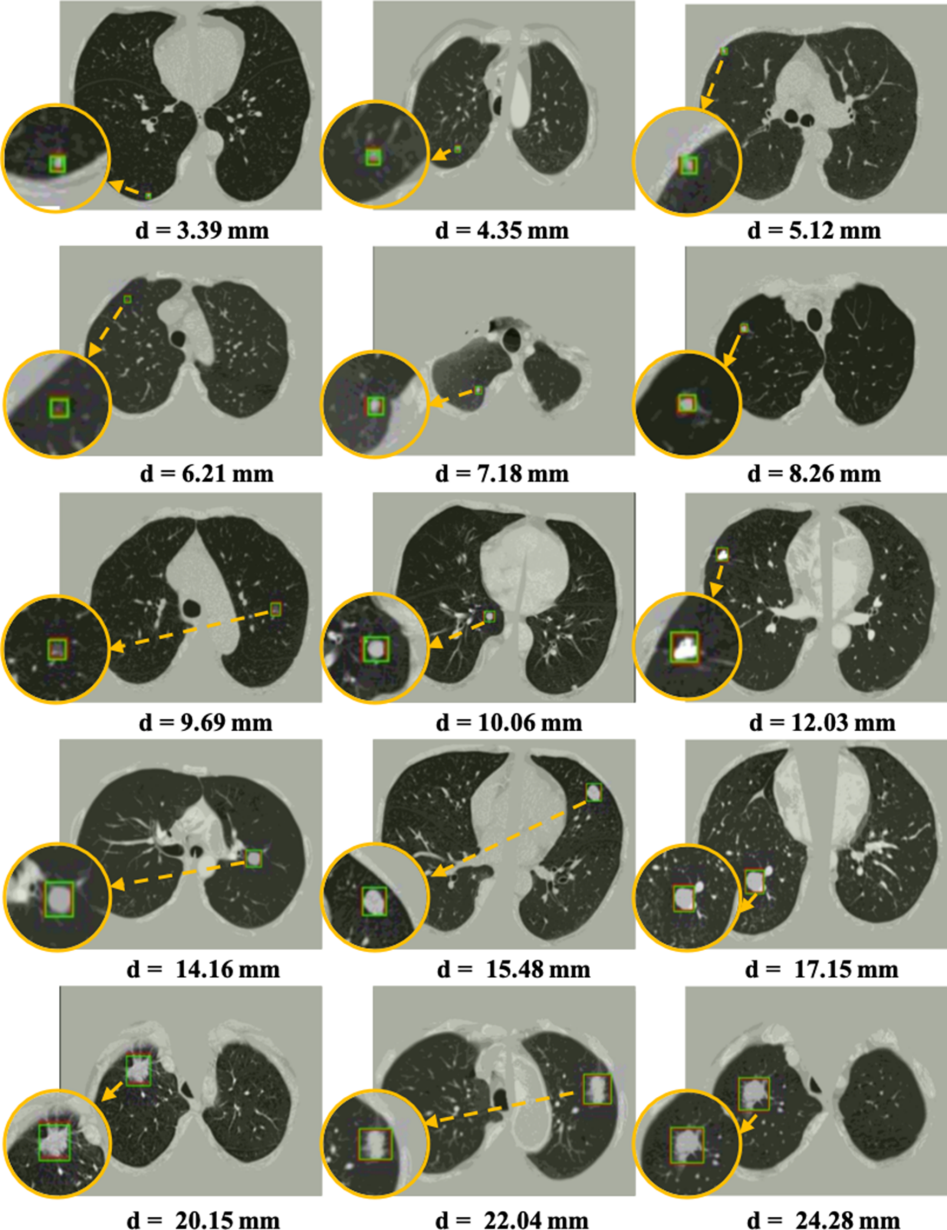
Visualization of some detected true nodules with different sizes from 3 mm to 25 mm in diameter d by our proposed *3DFPN-HS${}^{2}$* framework. For better visualization, the detected nodule regions are zoomed in, as shown in the orange circles. The green box indicates the predicted region, and the red box represents the ground-truth. Some of the red boxes are not observed because they are perfectly overlapped with the green boxes. The results demonstrate that our *3DFPN-HS${}^{2}$* framework is capable of detecting lung nodules of different sizes from CT scans accurately.

Table 4 shows the FROC evaluation results at 1/8, 1/4, 1/2, 1, 2, 4, 8 false-positive levels for our proposed method compared with state-of-the-art methods on LUNA16. The highlighted numbers in the table represent the best performance for each column. Since most state-of-the-art methods do not use the pre-trained model, all the methods are tested on the LUNA16 dataset without the pre-trained model for a fair comparison, following the same FROC evaluation. The state-of-the-art methods are divided into two groups for the frameworks without false positive reduction (14,16,41) and with false positive reduction process (12,13,15,42,43). As shown in the table, compared with the state-of-the-art detection methods, the proposed *3DFPN* surpasses 13.9% false positive at 0.125 per scan and 2% on average CPM. Compared with the framework with the false positive reduction, our framework outperforms 5.5% average sensitivity over most results of other methods and 1% than Kim et al. (43). Moreover, the proposed framework achieves the best performance at most of FP levels. As mentioned above, CAD systems require not only high sensitivity but also high specificity. Table 4 shows that the proposed *HS${}^{2}$* network significantly reduces false positives. *3DFPN-HS${}^{2}$* obtains the maximum sensitivity of 97.14% for 2 FPs per scan. Additionally, the proposed framework remains a high sensitivity above 90% for the 1/8, 1/4, and 1/2 FP per scan. The experimental results show that the proposed *3DFPN-HS${}^{2}$* reaches high sensitivity and specificity with state-of-the-art performance for lung nodule detection. The nodule detection results are shown in Figure 7.

**Table 4:** FROC Performance comparison with the state-of-the-art methods on LUNA16 dataset: sensitivity and the corresponding false positives at 1/8, 1/4, 1/2, 1, 2, 4, 8 per scan. Our *3DFPN-HS${}^{2}$* method achieves the best performance (with >90% sensitivity) at most of false positive levels and significantly outperforms others especially at the low false positive levels (1/8 and 1/4). *3DFPN* indicates the nodule detector without false positive reduction. *3DFPN-HS${}^{2}$* is with false positive reduction. *3DFPN-HS${}^{2}⋆$* shows the results applying pre-trained model trained on the NLST dataset. $CPM_{s}$,$CPM_{m}$, and $CPM_{l}$ show the average detection performance for the small, medium, and larger nodules, respectively.

Methods	1/8	1/4	1/2	1	2	4	8	CPM Score	$CPM_{s}$	$CPM_{m}$	$CPM_{l}$
Without False Positive Reduction
Gupta et al. (41)	0.531	0.629	0.790	0.835	0.843	0.848	0.856	0.763	–	–	–
Zhu et al. (16)	0.692	0.769	0.824	0.865	0.893	0.917	0.933	0.842	–	–	–
Khosravan et al. (14)	0.709	0.836	0.921	0.953	0.953	0.953	0.953	0.897	–	–	–
With False Positive Reduction
Dou et al. (13)	0.677	0.737	0.815	0.848	0.879	0.907	0.922	0.827	–	–	–
Dou et al. (42)	0.659	0.745	0.819	0.865	0.906	0.933	0.946	0.839	–	–	–
Wang et al. (15)	0.676	0.776	0.879	0.949	0.958	0.958	0.958	0.878	–	–	–
Ding et al. (12)	0.748	0.853	0.887	0.922	0.938	0.944	0.946	0.891	–	–	–
Kim et al. (43)	0.904	0.931	0.943	0.947	0.952	0.956	0.962	0.942	–	–	–
3DFPN (Ours)	0.848	0.876	0.905	0.933	0.943	0.957	0.970	0.919	0.894	0.909	0.937
3DFPN-HS${}^{2}$ (Ours)	0.904	0.914	0.933	0.957	0.971	0.971	0.971	0.952	0.914	0.945	0.977
3DFPN-HS${}^{2}⋆$ (Ours)	0.906	0.923	0.948	0.981	0.981	0.981	0.981	0.957	0.928	0.953	0.979

To further analyze the performance of the detected network for various nodule sizes, we followed (53) to classify the test set into three categories. According to the size distribution of pulmonary nodules, the average CPM for 10-fold cross-validation are evaluated on the nodule sizes between 3 mm to 5 mm (small), 5 mm to 10 mm (medium), and larger than 10 mm (large), respectively. As shown in Table 4, *3DFPN-HS${}^{2}$* shows improvements in sensitivity for small, medium and large-sized nodule diameters compared with *3DFPN*. Specifically, the *3DFPN* performs well in detecting large and medium-sized nodule diameters as easy to detect. With the pre-trained model and false-positive reduction, the result shows a 3% improvement on average CPM for the small nodule detection and yields the best performance overall.

#### 4.5.2. Robustness with the self-supervised pre-trained model

As shown in Table 5, we conduct two sets of experiments to assess the robustness of frameworks that does not apply the pretrained model and that employ the pretrained model. For the experiment without the pre-trained model, the *3DFPN-HS${}^{2}$* model is trained from scratch on the LUNA16 training set. The experiment with the pre-trained model applies the weights from the pre-trained model to initialize the model parameters, and then fine-tunes the model on the LUNA16 dataset, with the results highlighted in Table 5. The models are trained and fine-tuned only on LUNA 16 training set and further tested on the LUNA16 testing set and three different datasets (SPIE-AAPM, LungTime, and HMS Lung Cancer datasets) for cross-dataset validation. Additionally, we compare the experiments without and with the false positive reduction method. Using the pre-trained model shows slight improvements at all the false positive levels than those without the pre-trained model. Experiments performed on SPIE-AAPM, LungTime, and HMS Lung Cancer datasets with the nodule detector trained only on the LUNA16 dataset shows a significantly decreasing performance compared to the results on the LUNA16 testing set, especially for 1/8 false positive per scan. It is because LUNA16 has a relatively limited training set and cannot robust to other datasets. Compared with the model trained only on the LUNA16 dataset, the framework applying the self-supervised pre-trained model shows a significant improvement in all false positive levels on all of these datasets. Specifically, on 1/8 false positive per scan, the sensitivity is increased 7.4% on SPIE-AAPM, 13.5% on LungTIME, and 8.9% on HMS, respectively. For the 8 false positives per scan, the accuracy is comparable to that of LUNA16. The significant improvement in performance demonstrates the robustness of applying the pre-trained model across different datasets without additional annotations. Because the model is trained on the LUNA16 training set, the test results on the LUNA16 test set have already achieved great performance as shown in Table 5. Therefore, compared with the other three datasets, the performance of this model is only slightly improved on the LUNA16 test set with the pre-training model than without the pre-trained model.

**Table 5:** FROC Performance comparison with and without using the pre-trained model, with and without false positive reduction: sensitivity and the corresponding false positives at 1/8, 1/4, 1/2, 1, 2, 4, 8 per scan. The *3DFPN-HS${}^{2}$* is only fine-tuned (with the pre-trained model)/trained (without the pre-trained model) on LUNA16 training set and tested on LUNA16 test set. At all levels of false positives per scan, the sensitivities of the framework using a pre-trained model and false positive reduction significantly outperform the one without the pre-trained model and false positive reduction.

Datasets	Pre-trained	$HS^{2}$	1/8	1/4	1/2	1	2	4	8	CPM Score
LUNA16	no	no	0.848	0.876	0.905	0.933	0.943	0.957	0.970	0.919
	no	√	0.904	0.914	0.933	0.957	0.971	0.971	0.971	0.952
	√	√	0.906	0.923	0.948	0.981	0.981	0.981	0.981	0.957
SPIE-AAPM	no	no	0.764	0.791	0.839	0.873	0.895	0.914	0.923	0.857
	no	√	0.808	0.852	0.877	0.914	0.928	0.947	0.964	0.899
	√	√	0.823	0.866	0.891	0.933	0.957	0.966	0.966	0.914
LungTIME	no	no	0.775	0.816	0.839	0.874	0.924	0.937	0.956	0.874
	no	√	0.797	0.826	0.862	0.891	0.927	0.948	0.969	0.889
	√	√	0.814	0.837	0.874	0.899	0.931	0.952	0.974	0.897
HMS Lung Cancer	no	no	0.624	0.681	0.712	0.784	0.827	0.898	0.909	0.776
	no	√	0.657	0.703	0.746	0.805	0.846	0.912	0.927	0.799
	√	√	0.752	0.823	0.856	0.894	0.921	0.941	0.958	0.877

#### 4.5.3. Effectiveness of HS${}^{2}$ network for FP reduction

**Figure 8:**
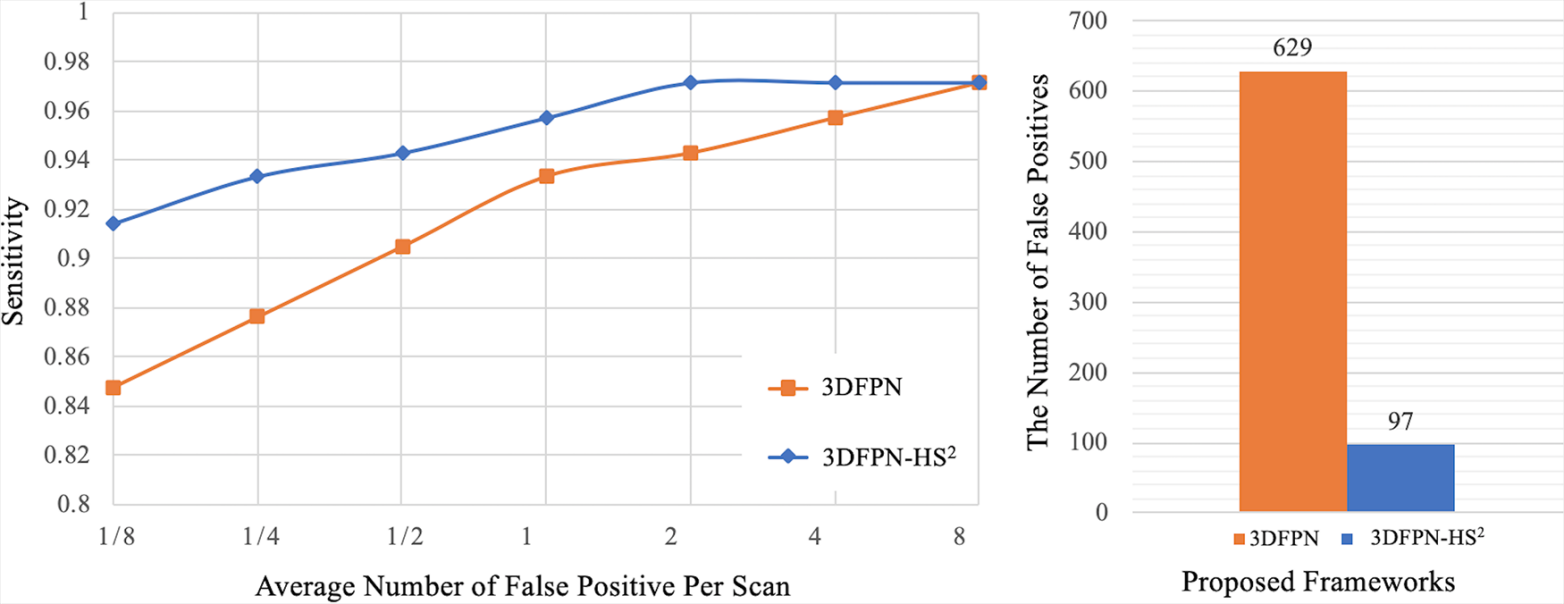
Comparison between the proposed *3DFPN* and *3DFPN-HS${}^{2}$*. Left: comparison of the proposed *3DFPN* and *3DFPN-HS${}^{2}$* on LUNA16 dataset without using the pre-trained model. *3DFPN-HS${}^{2}$* greatly improves the performance of the *3DFPN* at all FP levels per scan. Right: the number of false positives reduced from 629 to 97 for a total of 88 CT scans with the confidence score above 0 after applying the *HS${}^{2}$* network.

The superiority of *HS${}^{2}$* network on LUNA16 dataset is demonstrated by two experiments. As shown in Figure 8 (left), the result of *3DFPN-HS${}^{2}$* with the false positive reduction is increased more than 5% at 1/8 FP level compared with *3DFPN* without the *HS${}^{2}$* network. In addition, in Figure 8 (right), we further compare the numbers of FPs with (blue bar) and without (orange bar) *HS${}^{2}$* network in all the predicted nodule candidates in 88 CT scans (subset 9). The *3DFPN-HS${}^{2}$*, with *HS${}^{2}$* for false positive reduction, can distinguish the false detected tissues from true nodules, significantly reducing FPs by 84.5%. In addition, our proposed *3DFPN* without *HS${}^{2}$* network can still reach 97% at 8 FPs per scan, surpassing other state-of-the-art methods (shown in Table 4.)

## 5. Conclusion

In this paper, we have proposed an effective and robust *3DFPN-HS${}^{2}$* framework for lung nodule detection with a self-supervised feature learning schema. The different sizes of lung nodules can be detected by enriching the local and global features through a 3D feature pyramid network. By introducing the *HS${}^{2}$* network and treating each CT scan as a video, false positives are significantly reduced based on the patterns of location variance for nodules and tissues in continuous CT slices. Spatial features of CT scans can be effectively learned from large-scale CT scans without using additional labels by applying a self-supervised feature learning schema. The learned features can significantly improve the robustness of the proposed framework across different clinic datasets. As high sensitivity and specificity are achieved with robustness to the data from multiple CT scanner manufacturers, the proposed framework has a high potential in routine clinical practice.

## Data Availability

The original contributions presented in the study are included in the article/supplementary material, further inquiries can be directed to the corresponding author/s
